# Molecular Insights into Structural Dynamics and Binding Interactions of Selected Inhibitors Targeting SARS-CoV-2 Main Protease

**DOI:** 10.3390/ijms252413482

**Published:** 2024-12-16

**Authors:** Yuanyuan Wang, Yulin Zhou, Faez Iqbal Khan

**Affiliations:** Department of Biosciences and Bioinformatics, School of Science, Xi’an Jiaotong-Liverpool University, 111 Ren’ai Road, Suzhou 215123, China; yuanyuan.wang20@student.xjtlu.edu.cn (Y.W.); yulin.zhou20@alumni.xjtlu.edu.cn (Y.Z.)

**Keywords:** SARS-CoV-2, Bofutrelvir, Paxlovid, Selinexor, molecular interactions, artificial intelligence

## Abstract

The SARS-CoV-2 main protease (Mpro, also known as 3CLpro) is a key target for antiviral therapy due to its critical role in viral replication and maturation. This study investigated the inhibitory effects of Bofutrelvir, Nirmatrelvir, and Selinexor on 3CLpro through molecular docking, molecular dynamics (MD) simulations, and free energy calculations. Nirmatrelvir exhibited the strongest binding affinity across docking tools (AutoDock Vina: −8.3 kcal/mol; DiffDock: −7.75 kcal/mol; DynamicBound: 7.59 to 7.89 kcal/mol), outperforming Selinexor and Bofutrelvir. Triplicate 300 ns MD simulations revealed that the Nirmatrelvir-3CLpro complex displayed high conformational stability, reduced root mean square deviation (RMSD), and a modest decrease in solvent-accessible surface area (SASA), indicating enhanced structural rigidity. Gibbs free energy analysis highlighted greater flexibility in unbound 3CLpro, stabilized by Nirmatrelvir binding, supported by stable hydrogen bonds. MolProphet prediction tools, targeting the Cys145 residue, confirmed that Nirmatrelvir exhibited the strongest binding, forming multiple hydrophobic, hydrogen, and π-stacking interactions with key residues, and had the lowest predicted IC_50_/EC_50_ (9.18 × 10^−8^ mol/L), indicating its superior potency. Bofutrelvir and Selinexor showed weaker interactions and higher IC_50_/EC_50_ values. MM/PBSA analysis calculated a binding free energy of −100.664 ± 0.691 kJ/mol for the Nirmatrelvir-3CLpro complex, further supporting its stability and binding potency. These results underscore Nirmatrelvir’s potential as a promising therapeutic agent for SARS-CoV-2 and provide novel insights into dynamic stabilizing interactions through AI-based docking and long-term MD simulations.

## 1. Introduction

In 2019, the emergence of the novel coronavirus SARS-CoV-2 triggered an unprecedented global pandemic, resulting in millions of infections and substantial loss of life [1,2,3]. This crisis underscores the urgent need for effective therapeutic strategies to mitigate the severity and mortality rates of COVID-19 [4,5,6]. SARS-CoV-2 is characterized by its spike (S) protein, which is responsible for viral entry, and an RNA genome encoding more than 24 proteins is crucial for viral replication and pathogenicity [7,8,9,10]. Once inside the host cell, viral RNA is translated into large polyproteins that are subsequently cleaved by two key viral proteases: the main protease (3CLpro, also known as Mpro) and papain-like protease (PLpro) [11,12,13]. Most cleavages are facilitated by 3CLpro, and inhibiting 3CLpro prevents the virus from producing the proteins necessary for replication [14,15,16]. Given its critical role in polyprotein processing and viral replication, 3CLpro has been identified as one of the most promising targets for antiviral drug development [14,15,17].

**Figure 1:**
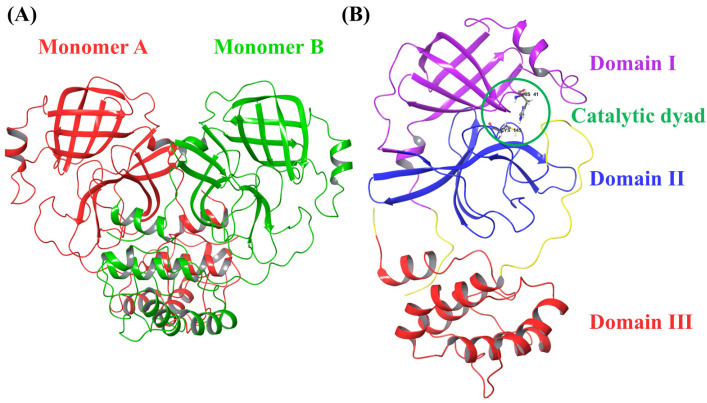
(**A**) Crystal structure of SARS-CoV-2 3CLpro (PDB: 1P9S) showing two monomers (monomer A in red and monomer B in green). (**B**) Crystal structure of the SARS-CoV-2 3CLpro monomer (PDB: 7ALH) showing the three domains.

3CLpro, a highly conserved enzyme in coronaviruses, plays a crucial role in cleaving viral polyproteins at several conserved sites, enabling successful viral replication [17,18,19]. This conservation makes 3CLpro a potential target for the design of broad-spectrum antiviral inhibitors that may be effective across different coronaviruses (Figure 1).

We have previously studied the molecular basis of the effect of temperature on the structure and function of the SARS-CoV-2 spike protein. Our findings revealed that higher temperatures have little or no influence on the stability and folding of the SARS-CoV-2 spike protein [20]. We have also explored inhibitors targeting key structural proteins of SARS-CoV-2, including the S protein, nucleocapsid (N) protein, and membrane (M) protein [20,21]. Additionally, compounds such as Remdesivir have demonstrated potent inhibitory effects on 3CLpro, RNA-dependent RNA polymerase (RdRP), and other viral components [22]. Natural compounds, such as *Withania somnifera* (Ashwagandha) and psilocybin mushroom, have been proposed for their potential therapeutic effects and clinical management of SARS-CoV-2 infection [1,23]. Despite these advancements, there is a need for a more detailed comparative research on orally available inhibitors that specifically target 3CLpro [24].

PF-07321332 (Nirmatrelvir), a key component of the oral antiviral drug Paxlovid, is a potent 3CLpro inhibitor with significant therapeutic potential against SARS-CoV-2 [25]. Several studies have explored the Nirmatrelvir-3CLpro complex using MD simulations, to identify stable interactions between Nirmatrelvir and the catalytic triad (Cys145–His41–Asp187) of 3CLpro, and confirm the complex’s conformational stability through reduced structural fluctuations in the Nirmatrelvir-bound state [26,27,28]. Additionally, research on active-site mutations within 3CLpro, such as C145A and C145S, demonstrated significant reductions in Nirmatrelvir’s binding free energy and complex structural destabilization, underscoring the challenges posed by emerging drug resistance [29,30]. These in silico analyses provide valuable insights into the binding affinity and structural dynamics of the Nirmatrelvir-3CLpro complex, which are crucial for optimizing drug design. However, few studies have performed comparative analyses of different compounds targeting 3CLpro, particularly using advanced computational methods and long-term MD simulations to evaluate binding stability over extended time scales.

In this study, we employed AI-based docking tools powered by machine learning algorithms to predict the optimal binding of drug candidates to 3CLpro. Additionally, we performed long-term MD simulations to create a dynamic, time-resolved model of the molecular interactions. Our focus is on investigating the inhibitory effects of the first three oral medications, Nirmatrelvir, Bofutrelvir, and Selinexor, which have entered clinical trials (Table 1) and target 3CLpro. By evaluating their binding efficiency and inhibitory potential, we aim to identify the most promising candidates for targeted antiviral therapy, ultimately contributing to more effective treatment options for COVID-19.

## 2. Results

### 2.1. Molecular Interactions

Molecular docking is an essential tool in drug discovery, as it predicts the binding orientation of small molecules to their protein targets, facilitating the identification of potential inhibitors [31]. In this study, the binding affinities of Nirmatrelvir, Selinexor, and Bofutrelvir to SARS-CoV-2 3CLpro were evaluated by molecular docking, specifically targeting the active-site residue Cys145. Cys145 is part of the catalytic dyad (Cys145–His41), which is essential for the proteolytic activity of 3CLpro [32]. Targeting Cys145 effectively inhibits protease activity, thereby preventing viral replication [33].

**Figure 2:**
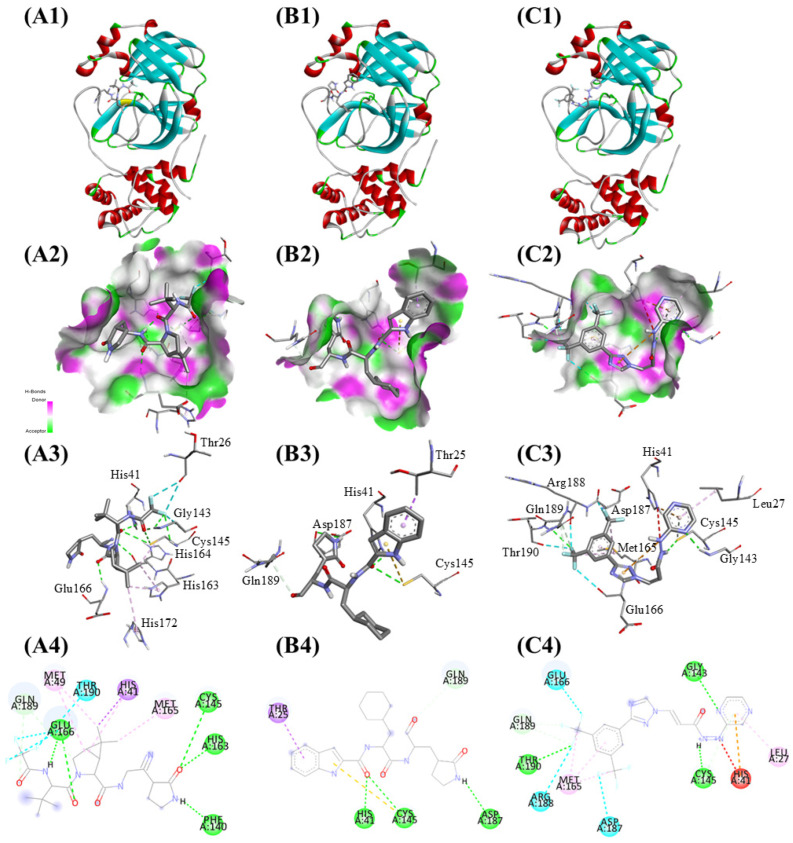
Binding interactions of the three compounds with SARS-CoV-2 3CLpro: (**A**) Nirmatrelvir, (**B**) Selinexor, and (**C**) Bofutrelvir. (**1**) Active site view showing drug binding. (**2**) Surface view highlighting hydrogen bond donors and acceptors. (**3**) Atom-level view of the interactions between the ligand and key active-site residues. (**4**) 2D interaction map including hydrogen bonds (green dashed lines), hydrophobic interactions (purple dashed lines), and unfavorable contacts (red dashed lines).

Nirmatrelvir demonstrated the best docking score with a binding affinity of −8.3 kcal/mol. Key hydrogen bonds formed with Phe140, Cys145, His163, and Glu166 contributed to stabilizing Nirmatrelvir within the binding site, with additional hydrophobic interactions involving His41 providing further stabilization. Selinexor exhibited a slightly lower binding affinity of −8.2 kcal/mol. It formed hydrogen bonds with His41, Cys145, and Asp187, along with hydrophobic contacts involving Thr25. These interactions anchored Selinexor within the active pocket, demonstrating moderate potential as an inhibitor. Bofutrelvir had the lowest binding affinity at −7.9 kcal/mol. Hydrogen bonds formed with Gly143, Cys145, and Thr190 were observed. However, a positive-positive charge repulsion with His41 likely contributed to its reduced binding affinity compared to the other compounds (Figure 2). These results indicate effective interactions of the three compounds with the viral protease, with Nirmatrelvir displaying the strongest binding affinity and stabilization potential, making it the most promising candidate for SARS-CoV-2 3CLpro inhibition (Table 2).

### 2.2. Ligand-Specific Protein Conformational Changes

The DiffDock analysis indicated a strong affinity of Nirmatrelvir, Bofutrelvir, and Selinexor for SARS-CoV-2 3CLpro, with SMINA docking scores of −7.75 kcal/mol, −6.95 kcal/mol, and −7.04 kcal/mol, respectively. Nirmatrelvir demonstrated the highest binding affinity among the three ligands, indicating the strongest interaction with SARS-CoV-2 3CLpro. In contrast, Bofutrelvir and Selinexor exhibited slightly lower affinities but remained promising candidates for 3CLpro inhibition, although they may not bind as strongly as Nirmatrelvir.

**Figure 3:**
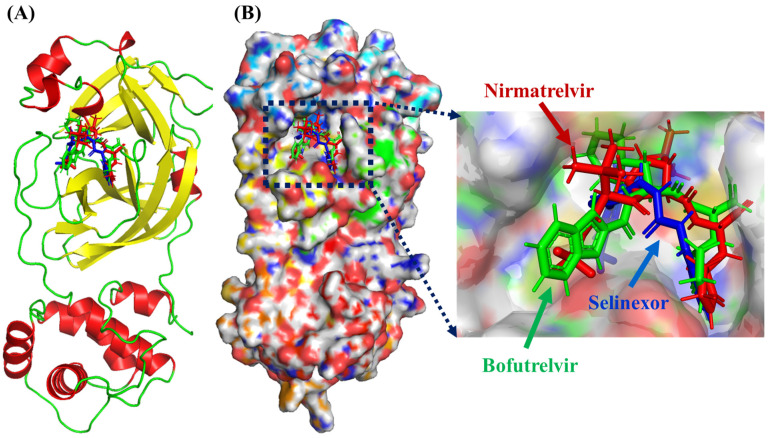
Deep equivariant generative model sampling. (**A**) The dynamic docking investigation of Nirmatrelvir (red), Bofutrelvir (green), and Selinexor (blue) into the active pocket of SARS-CoV-2 Mpro. In the protein structure, α-helices are shown in red, β-sheets in yellow, and loop regions in green. (**B**) Protein surface view showing the dynamic poses of these ligands.

The output of DynamicBound includes the rotation for both the ligand and each protein residue, rotation of torsional angles for the ligands, chi angles for the protein residues, and two prediction modules: binding affinity and confidence score (Figure 3). Nirmatrelvir consistently ranks high in terms of binding affinity (with values ranging from 7.59 to 7.89), indicating stable interaction. The confidence scores, ranging from 0.46 to 0.51, support the reliability of these predictions. Bofutrelvir shows good binding affinity as well (6.96 to 7.14) with slightly higher confidence scores (0.60 to 0.61) compared to Nirmatrelvir, suggesting a slightly more reliable prediction in terms of binding, even though its binding affinity is lower. Selinexor shows the lowest binding affinities (6.76 to 7.02) among the three, but its confidence scores are comparable to Nirmatrelvir’s (0.47 to 0.48), meaning the predictions are relatively reliable (Table 3).

### 2.3. MolProphet Analysis

It has been found that Nirmatrelvir, Bofutrelvir, and Selinexor can potentially form two, two, and four hydrogen bonds, respectively, with the Cys145 residue of SARS-CoV-2 3CLpro. Nirmatrelvir was found to have the lowest predicted IC_50_/EC_50_ activity of 9.18 × 10^−8^ mol/L and a binding efficacy index of 0.0141 (pIC_50/_MW). Bofutrelvir was found to have an IC_50/_EC_50_ activity of 2.64 × 10^−7^ mol/L and a binding efficacy index of 0.0145 (pIC_50/_MW). Selinexor was found to have an IC_50_/EC_50_ activity of 4.59 × 10^−7^ mol/L and a binding efficacy index of 0.0143 (pIC_50/_MW) (Table 4). MolProphet analysis revealed the potential of Nirmatrelvir, Bofutrelvir, and Selinexor to form hydrogen bonds with the Cys145 residue of SARS-CoV-2 3CLpro, with varying IC_50_/EC_50_ activities and binding efficacy indices. The predicted IC_50_/EC_50_ values (in mol/L) reflect the concentration of each drug required to inhibit 50% of the target activity (IC_50_) or achieve 50% of the desired effect (EC_50_). Lower IC_50_/EC_50_ values indicate higher potency, as the drug can achieve its inhibitory or therapeutic effects at lower concentrations. Nirmatrelvir has the lowest predicted IC_50_/EC_50_ value, meaning that it requires a much lower concentration to achieve 50% inhibition or effect compared to Bofutrelvir and Selinexor. This suggests that Nirmatrelvir is the most potent of the three, followed by Bofutrelvir and then Selinexor.

Nirmatrelvir can form six hydrophobic interactions and two π-stacking interactions with His41, one hydrophobic interaction with Met49, six hydrogen bonds with Asn142, three hydrogen bonds with His164, six hydrophobic interactions with Met165, 17 hydrogen bonds, two hydrophobic interactions with Glu166, one hydrogen bond with Asp187, two hydrogen bonds and seven hydrophobic interactions with Gln189, five hydrogen bonds and one halogen bond with Thr190, and four halogen bonds with Gln192 residues of SARS-CoV-2 Mpro.

Bofutrelvir can form two hydrogen bonds, one hydrophobic interaction, two π-stacking interactions with His41, six hydrophobic interactions with Met49, five hydrogen bonds with Asn142, six hydrophobic interactions with Met165, 14 hydrogen bonds and six hydrophobic interactions with Glu166, three hydrophobic interactions with Asp187, eight hydrophobic interactions with Gln189, and one hydrophobic interaction with Gln192 residues of SARS-CoV-2 Mpro.

Selinexor can form two hydrogen bonds and two π-stacking interactions with His41, five halogen bonds with Met49, seven hydrogen bonds with Asn142, three hydrogen bonds with His164, seven hydrophobic interactions with Met165, 12 hydrogen bonds, three halogen bonds with Glu166, two halogen bonds with Val186, seven halogen bonds with Arg188, five hydrophobic interactions with Gln189, three halogen bonds with Thr190, and six halogen bonds with Gln192 residues in SARS-CoV-2 Mpro.

**Figure 4:**
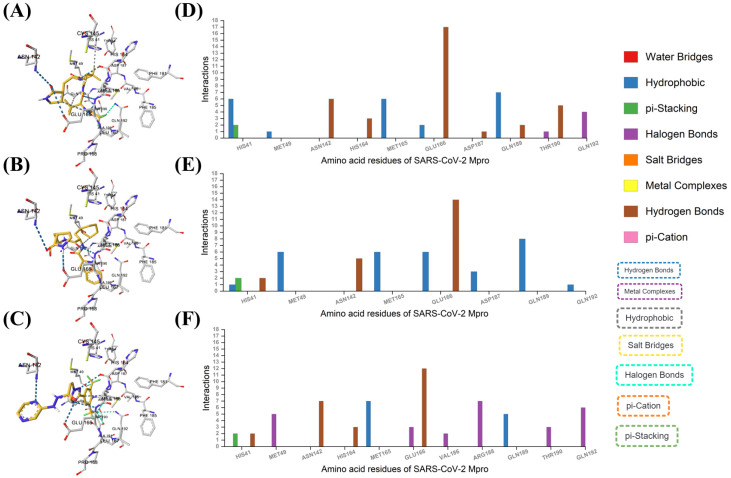
AI-based molecular interactions. The binding modes of (**A**) Nirmatrelvir, (**B**) Bofutrelvir, and (**C**) Selinexor with SARS-CoV-2 Mpro. Analysis of the interactions of SARS-CoV-2 Mpro residues with (**D**) Nirmatrelvir, (**E**) Bofutrelvir, and (**F**) Selinexor.

Based on the interactions described, Nirmatrelvir appears to be the most promising drug for inhibiting SARS-CoV-2 Mpro. It exhibits the highest number of interactions, including extensive hydrogen bonding (notably 17 with Glu166, a key residue), multiple hydrophobic interactions, and π-stacking interactions with critical residues like His41 and Gln189 (Figure 4). These strong and diverse interactions with essential residues of Mpro suggest that Nirmatrelvir may have a higher binding affinity and potentially more robust inhibitory effects compared to Bofutrelvir and Selinexor.

### 2.4. Structural Dynamics and Deviations

**Figure 5:**
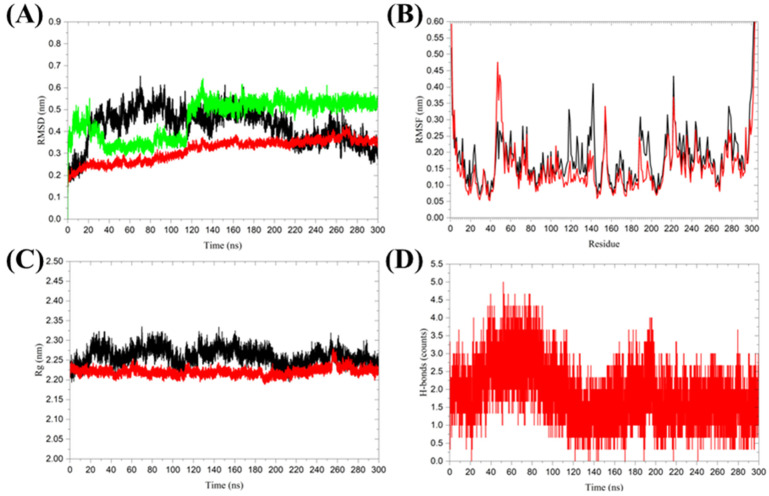
Molecular dynamics simulations of SARS-CoV-2 3CL protease in its Nirmatrelvir-unbound and Nirmatrelvir-bound forms. (**A**) Root mean square deviation (RMSD), (**B**) Root mean square fluctuations (RMSF), (**C**) Radius of gyration (Rg), and (**D**) Hydrogen bond analysis between Nirmatrelvir and 3CL protease. The color black represents 3CL protease alone, while red indicates the 3CL protease in the Nirmatrelvir-3CL protease complex, and green represents the ligand in the Nirmatrelvir-3CL protease complex. The presented charts are the average representation of the triplicate MD simulation runs for the Nirmatrelvir-3CL protease complex.

MD simulations provide insights into the dynamic behavior of biomolecular complexes [20], allowing researchers to understand conformational changes [34], stability [35], point mutations, and the effects of environmental conditions on protein-ligand interactions [36]. By combining molecular docking and MD simulations, researchers can refine binding poses, evaluate binding affinities, and predict long-term stability of drug candidates in biological systems. Structural dynamics were studied through triplicate 300 ns MD simulations for Nirmatrelvir-bound 3CLpro and a single 300 ns simulation for unbound 3CLpro to investigate the detailed binding effects of Nirmatrelvir on the 3CL protease (Table 5) [37,38]. The root mean square deviation (RMSD) is a crucial property for determining whether a protein is stable and resembles its experimental structure [39]. The RMSD analysis revealed distinct stability profiles for the Nirmatrelvir-unbound and Nirmatrelvir-bound forms of 3CLpro. The 3CLpro, in its unbound form, showed a higher average RMSD of 0.424 ± 0.080 nm, reflecting larger fluctuations in its structural conformation. In contrast, the 3CLpro in the Nirmatrelvir-bound complex exhibited a lower average RMSD of 0.315 ± 0.051 nm, stabilizing after approximately 120 ns, suggesting that Nirmatrelvir binding enhances the conformational stability of 3CLpro. The ligand RMSD showed an average value of 0.464 ± 0.086 nm, with initial fluctuations that gradually converged to a stable conformation around 0.55 nm after 120 ns (Figure 5A). The root mean square fluctuations (RMSF) analysis, a commonly used method to assess protein flexibility [40], revealed distinct fluctuation patterns between the Nirmatrelvir-unbound and Nirmatrelvir-bound forms of 3CLpro. In the unbound form, residues 118–143, particularly those within the loop regions, exhibited high fluctuations, indicating structural flexibility. In the Nirmatrelvir-bound complex, residues 45–56, located near the Nirmatrelvir binding site, displayed increased fluctuations. This localized flexibility in the binding region may facilitate effective ligand binding and interaction (Figure 5B). These results underscore the importance of understanding the molecular interactions between Nirmatrelvir and 3CL protease, which are critical for the development of targeted therapeutic strategies against SARS-CoV-2.

The radius of gyration is an indicator of the compactness of a protein structure [41]. The average Rg for 3CLpro in the Nirmatrelvir-bound complex was 2.221 ± 0.010 nm, which is slightly lower than the 2.260 ± 0.020 nm observed in the Nirmatrelvir-unbound form. Although the difference in average Rg values between the two forms was modest, Nirmatrelvir binding significantly reduced Rg fluctuations, indicating the enhanced structural stability of the 3CL protease (Figure 5C). Hydrogen bond analysis is a widely used method for studying protein-ligand interactions [42]. During the 300 ns MD simulation, Nirmatrelvir formed an average of 2 ± 1 hydrogen bonds with 3CLpro (Figure 5D). This suggests that Nirmatrelvir remains consistently bound to the active pocket of 3CLpro throughout the simulation. The formation of multiple hydrogen bonds indicates strong and stable interactions between Nirmatrelvir and 3CL protease, which may contribute to its high binding affinity and inhibitory potency.

### 2.5. Solvent-Accessible Surface Area

**Figure 6:**
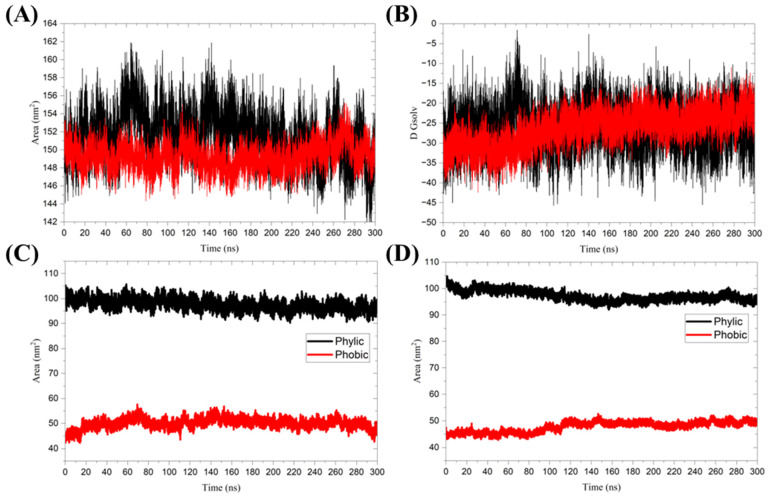
(**A**) Solvent-accessible surface area, and (**B**) Free energy of solvation. The color black represents 3CL protease alone, while red indicates the 3CL protease in the Nirmatrelvir-3CL protease complex. The SASA was further divided into hydrophobic and hydrophilic regions for (**C**) 3CL protease alone and (**D**) 3CL protease in the Nirmatrelvir-3CL protease complex. The presented charts are the average representation of the triplicate MD simulation runs for the Nirmatrelvir-3CL protease complex.

The solvent-accessible surface area (SASA) of a protein refers to the region that interacts with solvent molecules [43]. The mean SASA values for the 3CL protease in its unbound form and the Nirmatrelvir-bound complex were 151.6 ± 2.7 nm^2^ and 149.3 ± 1.4 nm^2^, respectively. The slight reduction in SASA upon ligand binding suggests that the protein becomes less solvent-accessible, indicating the stabilization of the binding region. Additionally, Nirmatrelvir was observed to be binding resulted in fewer SASA fluctuations of the 3CL protease compared to the unbound form (Figure 6A). This further indicates that the binding of Nirmatrelvir restricts the conformational flexibility of the 3CLpro, thereby stabilizing complex formation. This modest reduction in SASA, combined with a stable Rg and increased RMSF of active-site residues, suggests that Nirmatrelvir induces localized conformational changes primarily near the binding site, rather than causing a global rearrangement of the protease structure. These localized changes may play a critical role in stabilizing the protein-ligand complex and enhancing binding affinity.

The free energy of solvation (ΔG_solv_) measures the change in free energy required to transfer a protein from a vacuum to a solvent environment. A more negative ΔG_solv_ indicates stronger interactions between the protein and the solvent, reflecting higher solubility [44]. The average ΔG_solv_ for the unbound 3CLpro is −26.07 ± 5.32 kcal/mol, while the ΔG_solv_ for the 3CL protease in complex with Nirmatrelvir is −26.34 ± 4.34 kcal/mol (Figure 6B). The small difference in ΔG_solv_ between the ligand-unbound and ligand-bound forms of 3CL protease suggests that Nirmatrelvir binding does not significantly impact the global solvation properties of the protein. The SASA was further divided into hydrophobic and hydrophilic regions for unbound 3CLpro and 3CLpro in the Nirmatrelvir-3CL protease complex (Figure 6C,D). The hydrophobic regions of the 3CL protease exhibited a mean SASA of 50.030 ± 1.997 nm^2^ in the unbound form and 47.806 ± 1.927 nm^2^ in the Nirmatrelivir-bound complex. In contrast, the hydrophilic regions showed a mean SASA of 97.618 ± 2.167 nm^2^ for the unbound 3CLpro and 97.258 ± 1.918 nm^2^ for the Nirmatrelivir-bound form. Both hydrophobic and hydrophilic regions demonstrated reduced fluctuations upon ligand binding, indicating enhanced stability and minimized conformational variability in these regions when Nirmatrelivir is bound. Table 6 summarizes these contributions, along with the corresponding ΔG_solv_ values, highlighting a modest overall reduction in SASA upon ligand binding. A key distinction exists between the global average deviation for the triplicate simulations and the deviations observed within each individual replicate. The global average deviation provides an overall trend, indicating reduced SASA fluctuations in the bound form, as shown in Figure 6. In contrast, the individual replicate deviations reflect the inherent variability present within each simulation run, resulting in higher deviation values, as shown in Table 6. This distinction ensures a comprehensive understanding of both the general stability trends and the specific fluctuations observed across different simulation replicates.

### 2.6. Secondary Structure Analysis

**Figure 7:**
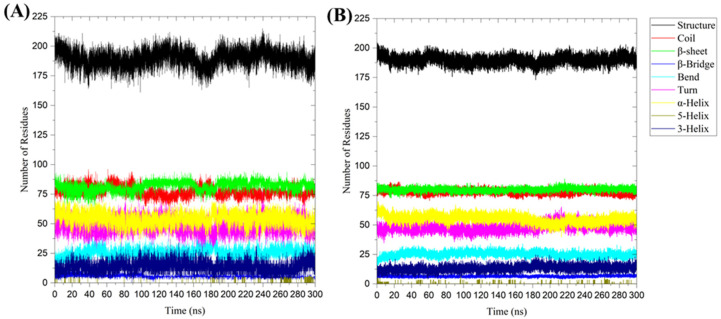
Secondary structure analysis during the 300 ns MD simulation for (**A**) unbound 3CL protease and (**B**) 3CL protease in the Nirmatrelvir-3CL protease complex. The presented charts are the average representation of the triplicate MD simulation runs for the Nirmatrelvir-3CL protease complex. Structure = α-helix + β-sheet + β-bridge + Turn.

The average number of residues involved in the formation of secondary structures in the 3CL protease was observed and compared between the Nirmatrelvir-unbound 3CLpro system and the Nirmatrelvir-3CL protease complex during the 300 ns MD simulations (Figure 7). The residues contributing to secondary structure formation were found to be 190 ± 7 in the 3CL protease alone and 190 ± 6 in the Nirmatrelvir-3CL protease complex, indicating that the overall folding and secondary structure elements are largely preserved regardless of ligand binding (Table 7). The similar secondary structure content of 3CLpro in its Nirmatrelvir-unbound and Nirmatrelvir-bound forms suggests that Nirmatrelvir binding induces localized structural rearrangements rather than global changes, thereby allowing strong binding without significantly altering the overall structure of 3CLpro.

### 2.7. PCA and GFE Landscape

**Figure 8:**
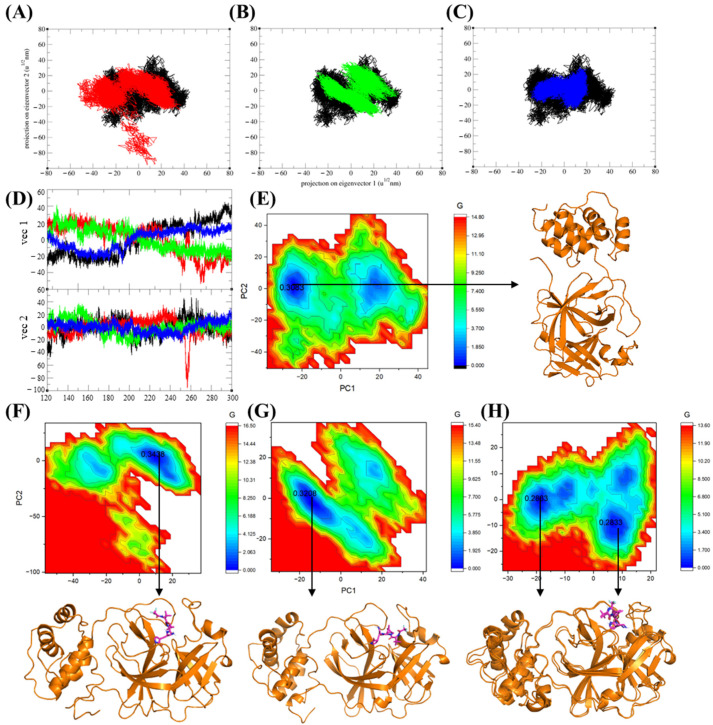
Principal component analysis (PCA) trajectory projections of 3CLpro along eigenvector 1 (PC1) and eigenvector 2 (PC2), comparing the Nirmatrelvir-unbound form and Nirmatrelvir-bound complexes. Trajectory projections for (**A**) replicate 1, (**B**) replicate 2, and (**C**) replicate 3. (**D**) Eigenvector analysis of the 3CLpro in its Nirmatrelvir-unbound form and Nirmatrelvir-bound complexes. The black color represents the Nirmatrelvir-unbound 3CL protease, while red, green, and blue correspond to the complexed 3CL protease in replicates 1, 2, and 3, respectively. (**E**) Gibbs free energy (GFE) landscape and the representative structure with the lowest free energy of the Nirmatrelvir-unbound 3CL protease, and (**F**–**H**) GFE landscapes and the representative structures with the lowest free energy of the 3CL protease in the Nirmatrelvir-3CL protease complex for replicates 1, 2, and 3. The numbers 0.3083, 0.3438, 0.3208, and 0.2833 represent the free energy values (in kcal/mol) of the minima energy basins, indicating the most stable conformational states within the energy landscape.

Principal component analysis (PCA) is a widely used statistical technique that reduces the dimensionality of high-dimensional data sets [45]. In protein dynamics, PCA is particularly useful for identifying clusters and examining significant collective motion within a protein molecule. We employed MD trajectories from 120 ns to 300 ns to perform PCA analysis for the Nirmatrelvir-unbound 3CL protease and the Nirmatrelvir-bound 3CL protease complex. The two-dimensional projection of the 3CL protease in the Nirmatrelvir-bound state revealed a restricted distribution in the conformational space compared to the unbound form. This indicates that Nirmatrelvir binding significantly stabilizes the movement patterns of the 3CL protease. The Nirmatrelvir-unbound form exhibited a broader and more diverse distribution, reflecting greater conformational freedom and flexibility in the absence of the ligand (Figure 8A–C). The sum of eigenvalues for the 3CL protease in its unbound form was 1410.21, while the 3CL protease in the Nirmatrelvir-3CL protease complex exhibited eigenvalues of 1371.17, 968.16, and 841.44 for replicates 1, 2, and 3, respectively. These reduced structural variances of 3CL protease in the Nirmatrelvir-bound complex reflected the stabilizing effect of ligand binding on the 3CL protease. Furthermore, the eigenvector locus of 3CL protease alone was higher than that of the Nirmatrelvir-3CL protease complex, reflecting the greater conformational flexibility of the unbound protease (Figure 8D). These results suggest that Nirmatrelvir binding restricts the conformational freedom of 3CL protease, thereby stabilizing the protein structure. Table 8 presents the percentage of variance explained by the principal components (PC1 and PC2) during the 120–300 ns MD simulations.

The Gibbs free energy landscape (GFE) was further analyzed, with projections along the first (PC1) and second (PC2) eigenvectors [46] to identify energy minima and their corresponding representative lowest-energy structures. A single minimum energy basin was observed for the 3CL protease in its Nirmatrelvir-unbound form and in the Nirmatrelvir-bound complexes for replicates 1 and 2. In contrast, analysis of replicate 3 revealed two distinct low-energy states, with structural analysis showing that these states adopted similar conformations (Figure 8E–H). Additionally, the 3CL protease alone exhibits a larger region of low free energy, suggesting that it is more flexible or dynamic when unbound. This flexibility may be reduced upon binding to Nirmatrelvir, as drug binding often stabilizes the specific conformation of the protein.

### 2.8. Nirmatrelvir-3CL Protease Binding Analysis

Clustering analysis can be used to group similar conformational states from molecular simulations, helping identify stable and representative structures of the system [47]. To analyze the protein-ligand interactions in the Nirmatrelvir-3CLpro complex, clustering of representative structures from the triplicates of the MD simulations (120 ns to 300 ns) was performed. A total of 35, 29, and 17 clusters were identified for replicates 1, 2, and 3, respectively, with the largest clusters comprising 8269, 10,350, and 9283 structures. These clusters provided insights into the dominant conformational states of the complex.

**Figure 9:**
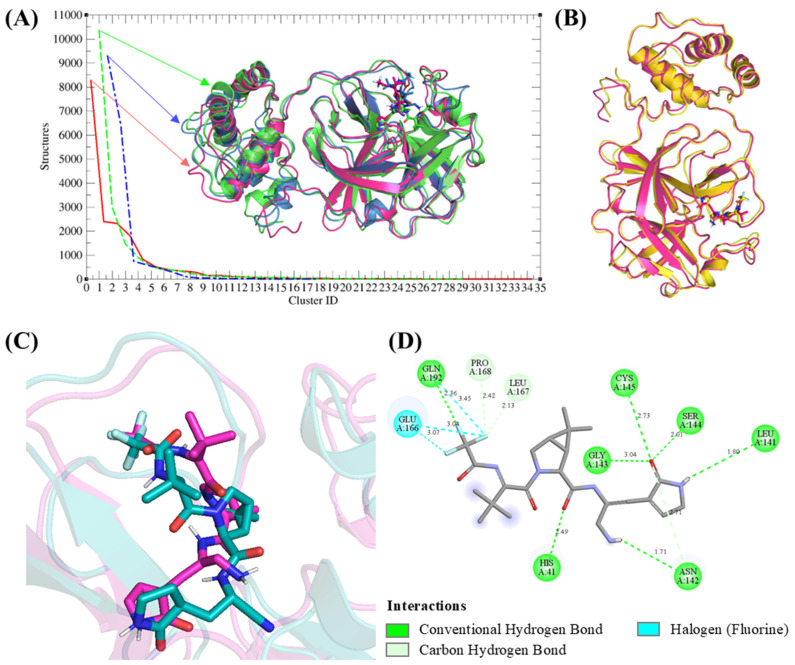
Clustering and binding analysis of the Nirmatrelvir-3CL protease complex. (**A**) Cluster analysis identified 35, 29, and 17 clusters for replicas 1, 2, and 3, respectively. (**B**) Superimposition of the representative structure of replica 1 (magenta) with its lowest-energy structure (yellow), showing close alignment. (**C**) Superimposition of Nirmatrelvir in replica 1’s representative structure (magenta) with its docked pose (teal), revealing minimal deviations. (**D**) 2D interaction analysis of Nirmatrelvir with 3CL protease based on the representative structure of replica 1.

Superimposition of the representative structures from the most populated clusters of all replicates revealed that Nirmatrelvir consistently adopted the same binding position in the active pocket of 3CL protease, with only minor deviations in orientation (Figure 9A). When comparing the representative structures of each replicate to their corresponding lowest-energy structures derived from Gibbs free energy analysis, we found that the representative structure from replica 1 aligned well with its lowest-energy structure, with an RMSD of 0.84 Å (Figure 9B). Consequently, a representative structure of replica 1 was selected for further binding analysis.

Nirmatrelvir maintained a consistent binding position and orientation compared to its initial docked pose, with minor deviations (2.486 Å) observed during MD simulations (Figure 9C). This consistency highlights the stability of Nirmatrelvir’s binding mode under dynamic conditions. Moreover, after MD simulations, Nirmatrelvir formed additional hydrogen and halogen bonds with key residues of the 3CL protease, enhancing its interactions compared to the docking results (Figure 9D). The increased interactions during MD simulations may be attributed to conformational flexibility within the binding pocket, allowing key residues to form additional stabilizing interactions. This flexibility likely enhances Nirmatrelvir’s binding stability by optimizing its orientation and aligning its functional groups with complementary sites in 3CL protease. These dynamic interactions reinforce the strong binding affinity observed for Nirmatrelvir in the simulations, providing further evidence of its potential as a robust inhibitor.

Binding free energy calculations for the Nirmatrelvir-3CLpro complex were performed during the stable segment of the trajectory (196–198 ns) in the MD simulation. This segment was specifically chosen because it corresponds to the stable conformation of 3CLpro, lying within the lowest energy well of the Gibbs free energy landscape. Extending these calculations to longer timescales or other regions with higher conformational variability would have introduced increased standard deviations, potentially reducing the reliability of binding energy estimates [48]. The van der Waals energy contributed significantly to this interaction, with a value of −144.659 ± 0.948 kJ/mol, indicating strong non-polar interactions between the Nirmatrelvir and the 3CLpro. The electrostatic energy contributed to a negative value of −9.009 ± 0.511 kJ/mol, further stabilizing the complex by promoting favorable electrostatic interactions. The polar solvation energy provided a positive contribution of 69.487 ± 0.970 kJ/mol, which reflects the tendency of solvent interactions to slightly destabilize the ligand-protein interactions. However, this destabilization effect was mitigated by the SASA (solvent-accessible surface area) energy, which contributed to a small negative value of −16.474 ± 0.121 kJ/mol, suggesting the stabilization of hydrophobic interactions within the complex. Altogether, the total binding energy for the 3CLpro-Nirmatrelvir complex was calculated to be −100.664 ± 0.691 kJ/mol, signifying a favorable binding interaction between the Nirmatrelvir and 3CLpro, primarily driven by van der Waals forces and supplemented by electrostatic interactions, with minor opposition from solvation effects.

## 3. Discussion

This study provides a comparative analysis of three protease inhibitors, Nirmatrelvir, Bofutrelvir, and Selinexor, targeting SARS-CoV-2 3CLpro through molecular docking and molecular dynamics simulations. Our results identified Nirmatrelvir as the most potent inhibitor, exhibiting the highest binding affinity (−8.3 kcal/mol) and forming multiple stable interactions with 3CLpro. Bofutrelvir and Selinexor also demonstrated effective binding but with slightly lower affinities (−7.9 kcal/mol and −8.2 kcal/mol, respectively). These findings provide valuable insights into the inhibitory mechanisms of these compounds and their potential roles in antiviral therapy.

Previous studies have explored various antiviral agents targeting different SARS-CoV-2 proteins. Our earlier research found that Remdesivir, which demonstrated a binding affinity of −7.8 kcal/mol to 3CLpro, could serve as an effective antiviral agent by targeting multiple viral components, including 3CLpro, RNA-dependent RNA polymerase (RdRP), and the membrane protein (M protein) of SARS-CoV-2 [22]. Natural compounds such as *fangchinoline* and *versicolactone C* have shown the potential to disrupt viral structural integrity by targeting key proteins such as the spike (S) protein, M protein, and nucleocapsid (N) protein [20,21]. However, the higher binding affinity of Nirmatrelvir, observed in this study, suggests that it may offer superior inhibition of the 3CLpro compared to other antiviral agents. This observation is consistent with Nirmatrelvir’s clinical efficacy in reducing viral load and symptom severity, as demonstrated in its approval for emergency use as part of the Paxlovid treatment.

Our results align closely with and build upon previous molecular dynamics (MD) studies of Nirmatrelvir in complex with 3CLpro, which consistently highlights Nirmatrelvir’s strong binding affinity and stability within the protease’s active site. Notably, previous studies have demonstrated that Nirmatrelvir forms robust interactions with critical catalytic residues, including Cys145 and His41, as well as with other nearby pocket residues, corroborating our findings [27,28,30,49]. Work by de Oliveira Só et al. also reported a reduction in the RMSD of the Nirmatrelvir-3CLpro complex during simulations, further supporting our observations of conformational stability [28]. Beyond RMSD, reductions in RMSF, Rg, and changes in hydrogen bonding patterns observed in our simulations align with previous reports of decreased protease flexibility upon Nirmatrelvir binding [28]. These findings not only corroborate Nirmatrelvir’s stabilizing effect on 3CLpro but also provide deeper insights into its influence on the protease’s structural dynamics. Moreover, our MD simulations reveal additional stabilizing interactions, including increased hydrogen bonding and halogen bonding interactions, which were not observed in earlier docking studies. This emphasizes the critical role of dynamic analyses in capturing ligand behavior and provides a framework for optimizing next-generation antiviral inhibitors targeting SARS-CoV-2 and other coronaviruses.

Molecular interaction analysis revealed that Nirmatrelvir had the strongest inhibitory effect, which was attributed to its ability to form multiple hydrogen bonds and hydrophobic interactions within the active site of 3CLpro, particularly with the catalytic residue Cys145. Deep equivariant generative model sampling and MolProphet molecular interactions also revealed that Nirmatrelvir emerges as the strongest candidate due to its higher binding affinity and reasonable confidence scores, supporting its known efficacy as a SARS-CoV-2 inhibitor. Bofutrelvir presents a promising option, with stable affinities and higher confidence scores than Nirmatrelvir, indicating good potential for further investigation. Selinexor shows moderate binding affinity and comparable confidence scores, suggesting that it may still be effective but might require additional optimization.

Molecular dynamics simulations revealed conformational stability in the Nirmatrelvir-3CLpro complex, as indicated by a modest reduction in root mean square deviation (RMSD). SASA analysis showed a slight decrease upon Nirmatrelvir binding, indicating a localized conformational change primarily near the binding site of the protease structure when complexed with this inhibitor. Nirmatrelvir also altered the Gibbs free energy landscape of the 3CLpro. The larger region of low free energy in the unbound 3CL protease suggests that it is more flexible and dynamic when unbound. This flexibility is reduced upon binding to Nirmatrelvir, as the inhibitor binding often stabilizes a specific conformation of the protease. Further, the formation of multiple hydrogen bonds throughout the simulations indicates strong and stable interactions between Nirmatrelvir and 3CLpro, which may contribute to its high binding affinity and inhibitory potency. We also identified the atomic coordinates corresponding to the lowest free energy for the Nirmatrelvir-bound 3CLprotease complex at a specific time: 186.200 ns for replica 1, 229.880 ns for replica 2, and 150.930 ns and 205.920 ns for replica 3. For the unbound 3CLprotease, the lowest free energy frame was observed at 125.900 ns. It is important to note that these conformations represent local stable states due to sampling limitations inherent in molecular dynamics rather than global minimum free-energy states. Further clustering analysis identified key interactions between Nirmatrelvir and the active-site residues, particularly the formation of a hydrogen bond with Cys145. This bond formation is complemented by additional interactions with nearby residues, further stabilizing the Nirmatrelvir-3CLpro complex.

Bofutrelvir, which shows a relatively lower binding affinity, remains a promising candidate due to its experimentally determined IC_50_ value of 53 nM [50] and its mechanism of targeting the same catalytic residues as Nirmatrelvir [50,51,52,53]. Although charge repulsion with His41 was observed in docking studies, clinical trials using inhalation delivery of Bofutrelvir suggest it could achieve high concentrations in the respiratory tract, which may enhance its therapeutic efficacy [51,52,53]. Selinexor, a selective nuclear export inhibitor [54], exhibited a comparable binding affinity to Nirmatrelvir, albeit slightly lower. Its ability to form hydrogen bonds with key residues, such as His41 and Cys145, despite being designed for other therapeutic uses, highlights its potential for repurposing as an antiviral agent. Although Selinexor’s primary mechanism differs from that of traditional protease inhibitors, its interaction with 3CLpro suggests that it may be a viable option for multi-target antiviral therapies.

While our computational study provides valuable insights into the inhibitory mechanisms of these compounds, the limitations of in silico methods must be acknowledged. Molecular docking and dynamic simulations offer a predictive model; however, the complexity of biological systems requires experimental validation. In addition, this study focused on noncovalent interactions to compare the binding energies of these inhibitors. Future studies should focus on in vitro and in vivo assays to confirm the inhibitory potency of these compounds and explore the pharmacokinetics and toxicity profiles that are not fully captured through computational methods.

## 4. Materials and Method

### 4.1. Molecular Docking

The molecular structures of three drugs (Nirmatrelvir, Bofutrelvir, and Selinexor) and the SARS-CoV-2 3CL protease were obtained from the PubChem database and the Protein Data Bank (PDB ID: 7C6S) [55], respectively. The SARS-CoV-2 3CL protease structure and drug structures (Nirmatrelvir, Bofutrelvir, and Selinexor) were prepared using AutoDock Tools version 1.5.7 (The Scripps Research Institute, La Jolla, CA, USA) [56], preprocessed by removing water molecules, adding hydrogen atoms, assigning Gasteiger charges, and minimizing energy for docking analysis. Cys145 is a key active-site residue and a nucleophilic target for SARS-CoV-2 Mpro inhibitors [57]. The configuration file was generated by defining a grid box centered on Cys145 with dimensions of 25 × 25 × 25 Å and a spacing of 1 Å, carefully adjusted to include adjacent active-site residues. The docking data were analyzed for protein-ligand complex stability, scoring function values, and molecular interactions. Important elements, such as hydrogen bonding, charge interactions, and hydrophobic interactions, were considered when assessing the stability and binding affinity of each protein-ligand complex.

### 4.2. Deep Equivariant Generative Model Sampling

To predict 3CL protease conformational changes upon binding of Bofutrelvir, Nirmatrelvir, and Selinexor, we employed the Neurosnap platform (Neurosnap Inc.—Computational Biology Platform for Research. Wilmington, DE, USA, 2022. https://neurosnap.ai/, accessed on 10 October 2024). This web-based bioinformatics tool integrates advanced computational biology algorithms, including DiffDock and DynamicBound, into a user-friendly interface. DiffDOCK (https://neurosnap.ai/service/DiffDock-L, accessed on 10 October 2024), a diffusion generative model for molecular docking, was used to dock Nirmatrelvir, Bofutrelvir, and Selinexor into the active pocket of SARS-CoV-2 Mpro [58]. DiffDOCK applies a diffusion process over the ligand pose transformation manifold to achieve accurate and efficient docking results, providing a novel approach for studying ligand-protein interactions. As part of its evaluation process, DiffDock integrates SMINA, a scoring function used to calculate the affinity score of docked ligand poses [59]. Additionally, DynamicBind (https://neurosnap.ai/service/DynamicBind, accessed on 10 October 2024), a deep learning method that uses equivariant geometric diffusion networks, was employed to construct a smooth energy landscape and facilitate efficient transitions between equilibrium states during docking [60]. It captures ligand-specific protein conformational changes and handles diverse protein dynamics, offering valuable insights into protein-ligand interactions.

### 4.3. MolProphet-Based Compound Targeting

Artificial intelligence (AI) is an effective tool for accelerating drug discovery and reducing costs during the discovery process. The amino acid Cys145 was targeted for molecular interactions of Nirmatrelvir, Bofutrelvir, and Selinexor with SARS-CoV-2 Mpro using the AI-based docking tool MolProphet [61]. It performs a rapid evaluation of a molecular data set through AI technology. Based on geometric deep learning, it learns target pocket information and small molecule structure information, and reinforcement learning is used to sample the receptor flexible conformation while optimizing the binding conformation of the ligand molecule to predict the minimum free energy of molecule binding to the target pocket.

### 4.4. Molecular Dynamics Simulation

The best docking pose for the Nirmatrelvir-3CLpro complex was used as the initial structure for MD simulations in GROMACS version 2023.2 [62,63]. The ligand parameters and topology files were generated using the ATB server (https://atb.uq.edu.au/, accessed on 29 March 2024). The protein-ligand complex was solvated in a cubic box with a 10 Å buffer filled with TIP3P water molecules. To neutralize the system and mimic physiological conditions, Na⁺ and Cl^−^ ions were added using the *gmx genion* module to achieve an ionic concentration of 0.15 M. Energy minimization was then performed using the steepest descent algorithm for 50,000 steps. Equilibration was conducted in two phases: first, the system was heated from 0 K to 310 K over 100 ps using the NVT ensemble at constant volume and temperature, with separate temperature coupling groups for the protein-ligand complex and water_and_ions. During this phase, position restraints with a force constant of 1000 kJ/(mol nm^2^) were applied to the protein and ligand using restraint files and LINCS constraints were used to stabilize all bonds involving hydrogen. Next, 100 ps of equilibration was performed under the NPT ensemble at constant pressure (1 bar) and temperature (310 K) using a V-rescale thermostat for temperature coupling and a Berendsen barostat for pressure coupling to stabilize the density and pressure while maintaining the same restraints and constraints. These restraints were removed before the production phase. Production MD simulations were conducted for 300 ns at 310 K, utilizing the Particle Mesh Ewald (PME) method for long-range electrostatics. A time step of 2 fs was used, and 30,001 frames were saved throughout the 300 ns production run for analysis. All simulations were carried out under the same conditions to ensure system stability, and the resulting trajectories were used for further analysis. We employed root mean square deviation (RMSD) to quantify structural differences between experimental and simulated protein structures, and we also used Root Mean Square Fluctuation (RMSF) to measure the displacement of specific atoms or groups relative to a reference structure. The radius of gyration (Rg) was used to evaluate the protein compactness and atomic distribution. Solvent-Accessible Surface Area (SASA) analysis was performed to assess the protein exposure to the solvent, providing insights into conformational changes and solvent interactions. The solvation-free energy (ΔG_solv_) was also calculated to quantify the energetic contribution of the solvent interactions. Principal Component Analysis (PCA) was employed for dimensionality reduction in large datasets to enhance interpretability. The Gibbs freeeEnergy (GFE) landscape was calculated to visualize the energetic distribution of the conformational states of the 3CL protease.

### 4.5. The Solvation-Free Energy (*ΔG_solv_*) Analysis

The solvation-free energy (ΔG_solv_) was calculated using the *gmx_mpi sasa* tool in GROMACS version 2023.2. The calculation is based on equation [64]:

(1) $$\Delta G\mathrm{solv}=\sum_{atoms\;i}\Delta \sigma iAi$$

where Δσi represents the atomic solvation parameter (ASP) of atom i. Ai represents the solvent-accessible surface area (SASA) of atom i. The *gmx_mpi sasa* tool calculates the SASA by modeling the surface area accessible to solvent molecules and outputs the solvation-free energy using the *-old* flag. This approach incorporates contributions from the hydrophilic and hydrophobic regions, with ASP values predefined in the GROMACS version 2023.2 library.

### 4.6. Principal Component Analysis

Principal Component Analysis (PCA) or Essential Dynamics (ED) was conducted for the 3CL protease alone and the 3CL protease-Nirmatrelvir complex by diagonalizing the covariance matrix C using the following equation:

C_ij_ = ⟨(r_i_ − <r_i_>) × (r_j_ − <r_j_>)⟩ (i, j = 1, 2, 3,…, 3N) (2)

Here, *r_i_* represents the Cartesian coordinate, i-th Cα atom, *N* denotes the number of Cα atoms, and <*r_i_*> indicates the time average for all configurations.

To ensure that the PCA focused on the intrinsic conformational changes of the 3CLpro, the trajectory was aligned to a reference structure using least squares fitting prior to the analysis. This alignment removed the effects of global translational and rotational motions. During the PCA analysis, the *gmx_mpi covar* tool was used, which automatically aligns all trajectory frames to the reference structure provided in the input file (md.tpr). This ensures that the covariance matrix reflects only the internal motions of the system. After diagonalizing the covariance matrix, *gmx_mpi anaeig* was utilized to analyze the eigenvectors and project the trajectory onto the first two principal components.

### 4.7. Gibbs Free Energy Landscape

The GFE landscape analysis can reveal potential structural and conformational alterations in both the 3CL protease and the 3CL protease-Nirmatrelvir complex. To visually represent the 2D and 3D conformations, the GFE landscape was projected onto PC1 and PC2 for the 3CL protease alone and the 3CL protease-Nirmatrelvir complex, respectively.

*G* _(PC1, PC2)_ = −*k*_B_*T* ln *P*_(PC1, PC2)_ (3)

**Figure 10:**
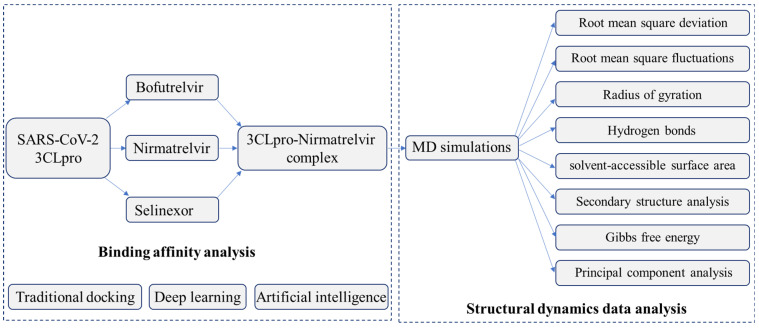
The flowchart outlining the analysis of binding affinity and structural dynamics of SARS-CoV-2 3CLpro and its inhibitors.

The *k*_B_, *T*, and *P*_(*PC1, PC2*)_ denote the Boltzmann constant, temperature, and normalized joint probability distribution for the 3CL protease alone and the 3CL protease-Nirmatrelvir complex, respectively. A flowchart outlining the data analysis is shown in Figure 10.

### 4.8. MM/PBSA Calculations

The binding free energy between 3CLpro and Nirmatrelvir was calculated using the MM/PBSA (Molecular Mechanics Poisson-Boltzmann Surface Area) approach. The molecular dynamics (MD) simulation trajectory was generated using GROMACS version 2023.2, and frames corresponding to the stable binding state of the ligand were extracted for analysis. The MM/PBSA calculations were conducted in three parts: the molecular mechanics (MM) component assessed van der Waals and electrostatic contributions, the Poisson-Boltzmann (PB) component evaluated the polar solvation energy, and the surface area (SA) component measured non-polar solvation via the solvent-accessible surface area (SASA). The final, binding free energy was calculated as the sum of these three components across multiple frames to capture the average interaction energy.

## 5. Conclusions

Our comparative analysis of Nirmatrelvir, Bofutrelvir, and Selinexor demonstrated that all three medications effectively inhibited the main SARS-CoV-2 protease. Notably, Nirmatrelvir, the active ingredient in Paxlovid, exhibited the highest binding affinity, highlighting its role as a potent inhibitor of 3CL protease. The binding of Nirmatrelvir not only stabilizes the 3CLpro but also induces localized structural rearrangements that likely disrupt enzymatic activity. Our findings support the potential of Nirmatrelvir as a valuable therapeutic agent against SARS-CoV-2, as it effectively targets critical viral enzymes necessary for the virus’s life cycle. This research underscores the importance of precise molecular interactions for the development of effective antiviral strategies.

## Figures and Tables

**Table 1:** Three drugs against the SARS-CoV-2 3CL protease and their detailed information.

Drug Name	Common Name	Design Team	FDA Approved	Clinical Trials	PubChem CID
Nirmatrelvir	Paxlovid	Pfizer	Yes	Yes	155903259
Bofutrelvir	FB2001	Shanghai Institute of Materia Medica, Chinese Academy of Sciences; Wuhan Branch, Chinese Academy of Sciences; Frontier Biotechnologies, Inc. (China)	No	Yes	145343771
Selinexor	KPT-330	Karyopharm Therapeutics	Yes	Yes	71481097

**Table 2:** The binding affinity of Nirmatrelvir, Selinexor, and Bofutrelvir.

Rank	Molecule	Affinity (kcal/mol)
1	Nirmatrelvir	−8.3
2	Selinexor	−8.2
3	Bofutrelvir	−7.9

**Table 3:** The ranking, binding affinity, and confidence score for the interactions between Nirmatrelvir, Bofutrelvir, Selinexor, and SARS-CoV-2 3CLpro.

Rank	Interaction	Binding Affinity	Confidence Score
1	Nirmatrelvir-SARS-CoV-2	7.89	0.51
2	Nirmatrelvir-SARS-CoV-2	7.59	0.46
3	Nirmatrelvir-SARS-CoV-2	7.71	0.46
1	Bofutrelvir-SARS-CoV-2	6.96	0.61
2	Bofutrelvir-SARS-CoV-2	7.14	0.60
3	Bofutrelvir-SARS-CoV-2	7.01	0.61
1	Selinexor-SARS-CoV-2	6.76	0.48
2	Selinexor-SARS-CoV-2	7.02	0.48
3	Selinexor-SARS-CoV-2	6.89	0.47

Notes: Binding Affinity: The binding affinity scores are calculated using the DynamicBound software (https://neurosnap.ai/service/DynamicBind, accessed on 10 October 2024), where higher positive values indicate stronger binding affinity. Confidence Score: The confidence score represents the reliability of the binding affinity prediction, with values closer to 1.0, indicating higher confidence in the prediction accuracy.

**Table 4:** The Predicted IC_50_/EC_50_ activity and binding efficacy index of Nirmatrelvir, Bofutrelvir, and Selinexor with SARS-CoV-2 Mpro.

S. No.	Drugs	Predicted IC_50_/EC_50_ Activity (mol/L)	Binding Efficacy Index (pIC_50_/MW)
1.	Nirmatrelvir	9.18 × 10^−8^	0.0141
2.	Bofutrelvir	2.64 × 10^−7^	0.0145
3.	Selinexor	4.59 × 10^−7^	0.0143

**Table 5:** Structural stability parameters for 3CL protease (Nirmatrelvir-unbound and Nirmatrelvir-bound forms) over 300 ns MD simulations.

System	Replica	RMSD (nm)	Ligand RMSD (nm)	RMSF (nm)	Rg (nm)	Hydrogen Bonds
3CL protease (Nirmatrelvir-unbound)	1	0.424 ± 0.080	-	0.192 ± 0.120	2.26 ± 0.020	-
3CL protease (Nirmatrelvir-bound)	1	0.351 ± 0.072	0.263 ± 0.068	0.179 ± 0.103	2.21 ± 0.023	2 ± 0.9
	2	0.284 ± 0.038	0.497 ± 0.233	0.156 ± 0.093	2.23 ± 0.014	2 ± 1.1
	3	0.310 ± 0.059	0.632 ± 0.056	0.148 ± 0.114	2.22 ± 0.012	1 ± 1.2

**Table 6:** Average SASA, ΔG_solv_, hydrophobic, and hydrophilic contributions to 3CLpro in its Nirmatrelvir-unbound and Nirmatrelvir-bound states.

System	Replica	SASA (nm^2^)	D G_solv_ (kcal/mol)	Phobic (nm^2^)	Phylic (nm^2^)
3CL protease (Nirmatrelvir-unbound)	1	151.593 ± 2.707	−26.072 ± 5.318	50.030 ± 1.997	97.618 ± 2.167
3CL protease (Nirmatrelvir-bound)	1	148.668 ± 2.797	−23.660 ± 6.092	48.251 ± 2.561	95.472 ± 2.876
	2	150.414 ± 2.467	−29.312 ± 5.362	46.481 ± 1.782	99.888 ± 1.991
	3	148.684 ± 2.348	−26.058 ± 6.904	48.686 ± 2.550	96.415 ± 2.646

**Table 7:** Average number of residues involved in secondary structure formation for 3CLpro in its Nirmatrelvir-unbound and Nirmatrelvir-bound forms during MD simulations.

System	Replica	Structure	Coil	β-Sheet	β-Bridge	Bend	Turn	α-Helix	5-Helix	3-Helix
3CL protease (Nirmatrelvir-unbound)	1	190 ± 7	77 ± 4	82 ± 4	6 ± 1	25 ± 4	48 ± 6	54 ± 5	0.1 ± 0.7	14 ± 5
3CL protease (Nirmatrelvir-bound)	1	190 ± 6	77 ± 4	80 ± 3	6 ± 1	27 ± 4	45 ± 5	59 ± 5	0.03 ± 0.4	13 ± 4
	2	190 ± 6	80 ± 3	80 ± 3	6 ± 1	25 ± 4	50 ± 6	53 ± 6	0.04 ± 0.5	12 ± 4
	3	190 ± 6	80 ± 3	80 ± 3	7 ± 1	24 ± 4	50 ± 6	54 ± 6	0.02 ± 1	15 ± 5

Note: Structure = α-helix + β-sheet + β-bridge + Turn.

**Table 8:** Percentage of variance explained by principal components (PC1 and PC2) during 120–300 ns MD simulations of 3CLpro in its Nirmatrelvir-unbound and Nirmatrelvir-bound forms.

System	Replica	PC1 Variance (%)	PC2 Variance (%)
3CL protease (Nirmatrelvir-unbound)	1	28.89	11.32
3CL protease (Nirmatrelvir-bound) 3CL protease-Nirmatrelvir	1	25.40	14.44
	2	18.58	15.29
	3	17.75	8.32

## Data Availability

The original contributions presented in the study are included in the article, and further inquiries can be directed to the corresponding author.
